# Poly(ε-L-lysine) and poly(L-diaminopropionic acid) co-produced from spent mushroom substrate fermentation: potential use as food preservatives

**DOI:** 10.1080/21655979.2022.2040876

**Published:** 2022-02-21

**Authors:** Mingxuan Wang, Chunchi Rong

**Affiliations:** a Institute of Food Science and Engineering, School of Health Science and Engineering, University of Shanghai for Science and Technology, Shanghai, Yangpu District, China; b School of Food Science and Pharmaceutical Engineering, Nanjing Normal University, Nanjing,Gulou, China

**Keywords:** Poly(ε-L-lysine), poly(L-diaminopropionic acid), spent mushroom substrate, co-production, food preservatives

## Abstract

Poly(ε-L-lysine) and poly(L-diaminopropionic acid) are valuable homopoly (amino acids) with antimicrobial properties and mainly produced in submerged fermentation. In this study, we investigated their co-production using waste biomass and spent mushroom substrate in solid-state fermentation. Simultaneous production of poly(L-diaminopropionic acid) and poly(ε-L-lysine) was achieved in a single fermentation process using pearl oyster mushroom residues as substrate, with the supplement of glycerol and corn steep liquor. After optimization of the fermentation parameters, the maximum yield of poly(ε-L-lysine) and poly(L-diaminopropionic acid) reached 51.4 mg/g substrate and 25.4 mg/g substrate, respectively. The optimal fermentation conditions were 70% initial moisture content, pH of 6.5, 30°C and an inoculum size of 14%. Furthermore, the fermentation time was reduced from 8 days to 6 days using repeated-batch solid-state fermentation. Finally, the antimicrobial effects of poly(L-diaminopropionic acid) and poly(ε-L-lysine) were evaluated in freshly pressed grape juice, which indicated tremendous potential of this mixture in its use as biological preservative.

## Graphical Abstract

**Figure:**
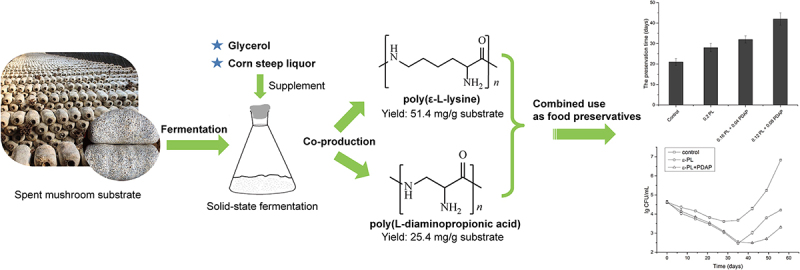

## 1. Introduction

Homopoly (amino acids) are unique biopolymers consisting of a single type of amino acids. In nature, only four homopoly (amino acids) are found poly(γ-glutamic acid) (γ-PGA), poly(L-diaminopropionic acid) (PDAP), poly(γ-L-diaminobutanoic acid) (γ-PAB) and poly(ε-L-lysine) (ε-PL) [1–3]. Homopoly (amino acids) secreted by microorganisms have been extensively studied, such as poly(ε-L-lysine), which has a broad antimicrobial spectrum, is used in food, medicines or new materials [4,5]. At present, poly(ε-L-lysine) is mainly produced industrially by fermentation using *Streptomyces albulus* [6]. Poly(L-diaminopropionic acid) is one of the secondary metabolites of *S. albulus* and is structurally similar to poly(ε-L-lysine). Despite their similarity, poly(L-diaminopropionic acid) has an antimicrobial spectrum that complements poly(ε-L-lysine), as it exhibits a stronger inhibitory effect on eukaryotic cells. Therefore, the combined use of poly(L-diaminopropionic acid) and poly(ε-L-lysine) could have tremendous potential in various fields. Although strategies involved in breeding of high-yield strains, pH control and dissolved oxygen regulation had been developed in submerged fermentation [7,8], co-production of the two valuable homopoly (amino acids) in solid-state fermentation has never been reported, and the cost of fermentation needs further reduction.

Solid-state fermentation (SSF) is regarded as a promising technology for the production of microbial secondary metabolites [9]. It produces high concentrations of product, consumes less energy, uses a variety of agricultural residues and provides a solid support for the mycelial morphology of microorganisms to produce secondary metabolites [10]. Recent examples of microbial products fermented on solid support including naphtho-gamma-pyrones (NγPs) in oil crop waste [11], lytic enzymes in wood waste [12] and iturin A in soybean meal and wheat grain [13]. On the other hand, the treatment of agricultural residues or waste biomass with microbes helps reduce environmental pollution and even recycle them into value-added products. For example, stale bread and brewers spent grain treated with edible filamentous fungi *Neurospora intermedia* and *Rhizopus oryzae* were converted into new protein-enriched products and re-introduced to food production chain [14]. And biological pretreatment with mixed microbes enhanced the degradation efficiency and biogas production using corn straw [15].

China produces more than 75% of worldwide edible fungus, yearly generating at least two million tons of spent mushroom substrate (SMS), a by-product of the mushroom industry that mainly consists of lignin, cellulose, hemicellulose and carbohydrates. Since spent mushroom substrate is not suitable for animal feed, treatment and disposal of this waste biomass is a major environmental problem. Therefore, the potential economic impact of recycling spent mushroom substrate is significant [16], and one way would be to bio-convert it into microbial products. Currently, spent mushroom substratesubstrates have been proposed as a source of biofuel and other biomaterials [17,18], and used in the production of industrially important metabolites by fermentation of fungi [19].

After evaluating the composition of residues from pearl oyster mushroom (*Pleurotus ostreatus*), we hypothesize that this spent mushroom substrate could be used for co-production of poly(L-diaminopropionic acid) and poly(ε-L-lysine) in solid state fermentation of *S. albulus*. The aim of this work was to recycle waste biomass spent mushroom substrates into high value-added homopoly (amino acids). In order to increase the yield of the two products, carbon and nitrogen sources were supplemented, and fermentation conditions were optimized. A repeated-batch process was also investigated to shorten the fermentation cycle. Finally, the extracted products were applied to freshly pressed grape juice as biological preservatives to evaluate their effects on food spoilage.

## 2. Materials and methods

### 2.1 Microorganisms and media

*S. albulus* CICC 11022 was purchased from the China Center of Industrial Culture Collection (CICC). The bevel medium contained 8 g/L glucose, 2 g/L polypeptone, 1 g/L yeast extract, 1 g/L beef meat extract, and 18 g/L agar. The medium used for the inoculum was composed of 50 g/L glucose, 10 g/L (NH_4_)_2_SO_4_, 5 g/L yeast extract, 0.5 g/L MgSO_4_ 7H_2_O, 0.04 g/L ZnSO_4_ 7H_2_O, 0.03 g/L FeSO_4_ 7H_2_O, 1.36 g/L KH_2_PO_4_ and 0.8 g/L K_2_HPO_4_. The pH of the medium was adjusted to 6.8 using NH_4_OH solution (25–28%, w/v). After complete sporulation on agar bevel medium, 100 mL medium in a 500 mL flask was inoculated with spore suspensions of *S. albulus* and cultured at 30°C with agitation at 200 rpm. This culture was used to inoculate the substrates in solid-state fermentation.

### 2.2 Analytical methods

The mushroom substrate residue was obtained from a local farm. The lignin content of the spent mushroom substrate was determined from the total solids and acid-insoluble lignin in biomass [20]. The cellulose and hemicellulose in spent mushroom substrate were hydrolyzed with 70% (w/w) sulfuric acid at 35°C for 1.5 h and the reaction mixture was diluted to 4% (w/w) sulfuric acid before autoclaving at 121°C for 1 h. This hydrolyzed solution was measured by an HPLC apparatus equipped with an Aminex column HPX-87 H (Bio-Rad, USA) [21]. For determination of soluble sugars, spent mushroom substrate was firstly boiled in water for 2.5 h and then phenol-sulfate was used in the supernatant according to a colorimetric method described previously [22]. And total nitrogen was determined after Kjeldahl digestion with concentrated sulfuric acid [23].

Dried spent mushroom substrates were heated in a Muffle furnace at 550°C, resulting in weight loss due to elimination of organic matter. To determine the mineral content, the ash from the Muffle furnace was dissolved in 50% HCl and 50% HNO_3_, filtered, and made up to 20 mL using distilled water. The phosphorous content was determined with a molybdate blue colorimetric method [24]. Elements were analyzed by atomic absorption spectroscopy in a Perkin Elmer Analyst AA200 [25], and all analyses were performed in triplicate.

For the analysis of fermentation products, a mixture of 2 g spent mushroom substrate and 20 mL distilled water was incubated for 1 h with stirring. The filtrate after cotton gauze was centrifuged at 9,000 g for 15 min at 4°C. Poly(L-diaminopropionic acid) and poly(ε-L-lysine) concentrations were determined from the supernatant by HPLC [26] using a TSKgel ODS-120 T column (Tosoh, Tokyo) equilibrated with 0.05% trifluoroacetic acid/acetonitrile (95:5) at 30°C. Absorbance was monitored at 215 nm, and flow rate was adjusted to 0.4 mL min^−1^. Firstly, poly(L-diaminopropionic acid) was eluted with equilibrating solution for 10 minutes and poly(ε-L-lysine) remained in the column. When the ratio of 0.05% trifluoroacetic acid to acetonitrile ranged from 85% to 95% in a linear gradient, ε-PL was eluted over 5 minutes. The yield of the two products was expressed as milligrams per gram of dry substrate (mg/g substrate). Residual glycerol in the supernatant was determined using HPLC with a differential refractometer (Agilent, 1200 series, USA) at 35°C, with flow rate of 0.5 mL/min and where the mobile phase was acetonitrile–water (90:10, v/v). Concentrations of glycerol was calculated based on calibration curves built using standard chemicals [27].

### 2.3 Solid-state fermentation and optimization of fermentation conditions

For the solid-state fermentation, 40 g of spent mushroom substrate was used in 500 mL flasks. After autoclaving at 121°C for 20 min, the substrates were inoculated with 8% of *S. albulus* liquid culture suspension and thoroughly mixed under aseptic conditions. For direct fermentation of spent mushroom substrate, fermentation was conducted at 30°C for 12 days. The yield of poly(L-diaminopropionic acid) and poly(ε-L-lysine) was determined by daily sampling. To screen suitable supplemental carbon sources in solid-state fermentation, wheat grain, rice bran, cane molasses, glycerol and corn flour were tested, and 4% glucose was used as a control. As nitrogen sources, we used 3% (w/w) of yeast extract, soybean meal, corn steep liquor and malt extract, whereas (NH_4_)_2_SO_4_ was used as control. All these supplemented raw materials were milled and sieved before addition. The fermentation temperature and time were 30°C and 8 days, respectively.

After optimizing the supplementary sources for solid-state fermentation, fermentation conditions were stepwisely optimized, and the optimization process was illustrated in Figure S1: initial pH, moisture content, fermentation temperature and initial inoculum size. The dried spent mushroom substrate was rehydrated to 55%, 60%, 65%, 70%, 75% or 80% moisture content. The pH was varied from 5.5 to 8.0. The incubation temperature was varied from 26°C to 36°C, and inoculum sizes were 5%, 8%, 11%, 14%, 17% or 20%.

### 2.4 Fermentation amplification and repeated -batch experiment

Amplified fermentation was conducted using 400 g of spent mushroom substrate in 5 L flasks using the optimized parameters: initial pH 6.5, 70% humidity, 14% inoculum size and 30°C fermentation temperature for 12 days. In repeated-batch fermentation, 10% substrate was used as an inoculum for subsequent batches and 8 cycles were conducted. For the determination of product concentration during the fermentation cycle, substrates were mixed, and 2 g samples were collected at 24 h intervals.

### 2.5 Extraction of products and food preservation experiment

A mixture of fermented spent mushroom substrate (50 g) and 500 mL distilled water was incubated for 1 h by stirring. The supernatant was filtered through cotton gauze and used for extraction of products. The pH of culture filtrate was first adjusted to 2.5 with HCl solution. For the extraction of poly(ε-L-lysine), the culture filtrate was saturated with methanol/acetone (3:1) from 40% to 67% and then purified according to methods previously described [28]. To obtain poly(L-diaminopropionic acid), the methods were modified in the precipitation step. Briefly, the supernatant was treated with 0% to 40% saturated methanol/acetone (3:1). The precipitate was redissolved in deionized water and subsequently purified using ion-exchange chromatography, ultrafiltration and reversed phase chromatography. Finally, poly(L-diaminopropionic acid) was freeze-dried resulting in a white powder. In the present study, the poly(ε-L-lysine) and poly(L-diaminopropionic acid) extracted was for experimental use only.

Good-quality grapes were purchased from the local market. After washing, cleaning and stemming, juice was extracted with an electric juicer and filtered with a muslin cloth. Freshly pressed grape juice was divided into different groups treated with 0.2 g/kg ε-PL, 0.16 g/kg ε-PL plus 0.04 g/kg PDAP, or 0.12 g/kg ε-PL plus 0.08 g/kg PDAP, respectively. Samples without treatment were used as a control. All the samples were stored at 4°C before organoleptic evaluation [29,30]. The total microorganism colonies in the control sample, samples treated with 0.2 g/kg ε-PL and sample treated with 0.12 g/kg ε-PL plus 0.08 g/kg PDAP were counted every week using a viable plate count method. Briefly, the samples were repeatedly diluted 10-fold into buffer and spread onto a plate counting agar medium. After the incubation at 37°C for 48 h, plates with 50 CFU to 150 CFU were used to calculate the total microorganism colonies. Each experiment was repeated three times.

## 3. Results and discussion

In this study, to recycle the waste biomass spent mushroom substrates into high value-added products, the composition of residues from pearl oyster mushroom (*Pleurotus ostreatus*) was analyzed, and we hypothesize that it was suitable for the growth of *S. albulus* and co-production of poly(L-diaminopropionic acid) and poly(ε-L-lysine) in solid state fermentation. To achieve this supplemental carbon and nitrogen sources were screened, fermentation conditions were optimized, and a repeated-batch process was investigated. Finally, the potential use of extracted products as food preservatives was evaluated in grape juice.

*3.1 Analysis of spent mushroom substrate for co-production of PDAP and ε-PL in solid-state fermentation*

The composition of *P. ostreatus* spent mushroom substrate was analyzed before bioconversion in solid-state fermentation, the results showed that it contained a high proportion of cellulose and hemicellulose (56.1%) and lignin (19.7%) in total weight. These structural macromolecules may provide an inert matrix within which carbon and nitrogen sources can be adsorbed and support mass and oxygen transfer [31], suggesting spent mushroom substrate from *P. ostreatus* could serve as a suitable solid substrate for the growth of *S. albulus*. The spent mushroom substrate contained 68.6% (w/w) moisture and the dried substrate contained 1.5% total nitrogen, 1.2% total soluble sugar and mineral elements (Table S1). Previous studies have shown that *S. albulus* required 50 g/L carbon and 10 g/L nitrogen for growth and metabolism, indicating that carbon and nitrogen content in spent mushroom substrates may not be sufficient to support the growth of *S. albulus* and the production of poly(L-diaminopropionic acid) and poly(ε-L-lysine) [7].

**Figure 1.**
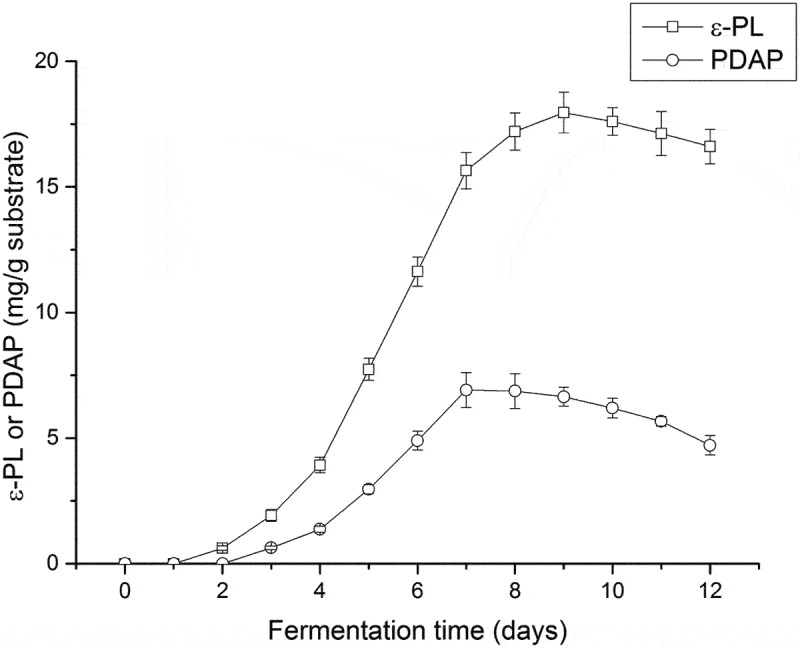
Fermentation using spent mushroom substrate without additives for the production of poly(L-diaminopropionic acid) (PDAP) and poly(ε-L-lysine) (ε-PL). Data points represent means (n = 3) ± standard deviation (SD).

Mineral elements play an important role in cell growth and metabolism [32] such as sodium, calcium, magnesium and iron are important in the biosynthesis of secondary metabolites by *S. albulus*. In this study, eight abundant mineral elements were detected in spent mushroom substrate from *P. ostreatus* (Table S1), the direct use of this substrate could be possible without additional mineral supplements in the fermentation. Thus, the spent mushroom substrate was initially tested without any additives, and the production of poly(L-diaminopropionic acid) and poly(ε-L-lysine) were recorded during 12 days of fermentation. The products poly(ε-L-lysine) and poly(L-diaminopropionic acid) were detected on the second and third day, respectively (Figure 1). When the yield of poly(ε-L-lysine) increased rapidly from the third day to the ninth day to 18.0 mg/g substrate, the accumulation of poly(L-diaminopropionic acid) appeared slower and the final yield was approximately one-third of the highest yield achieved for poly(ε-L-lysine). In the latter phase of fermentation, both metabolites decreased, possibly due to the depletion of nutrients in the substrate. These results suggest that solid fermentation of spent mushroom substrates by *S. albulus* is suitable for the production of both poly(L-diaminopropionic acid) and poly(ε-L-lysine). However, the direct use of spent mushroom substrate in solid-state fermentation is not advantageous in obtaining large quantities of products even though cellulase or other enzymes may be produced to increase the utilization of the substrate. Supplemental carbon and nitrogen sources are necessary since intracellular ATP levels are important for the biosynthesis of secondary metabolites [33].

*3.2 Screening of carbon sources and nitrogen sources for co-production of PDAP and ε-PL in solid-state fermentation using spent mushroom substrate*

**Figure 2.**
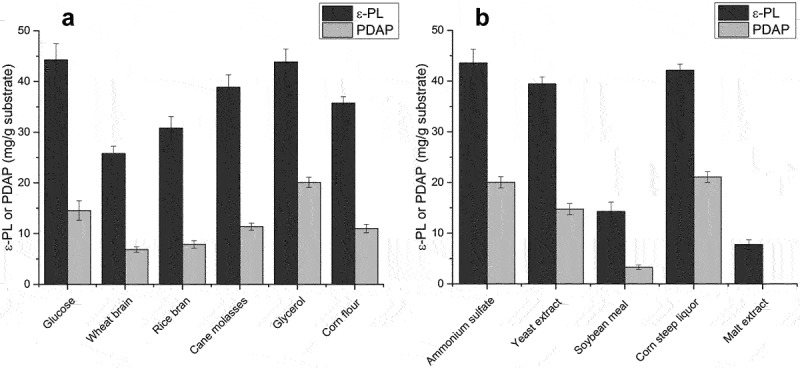
Determination of optimal carbon and nitrogen sources for solid-state fermentation supplementation. (a) Effect of different carbon sources on poly(L-diaminopropionic acid) (PDAP) and poly(ε-L-lysine) (ε-PL) production; (b) Effect of different nitrogen sources on PDAP and ε-PL production. Data points represent means (n = 3) ± standard deviation (SD).

Results of direct solid-state fermentation of spent mushroom substrate showed that the two homopoly (amino acids) could be co-produced in a single fermentation, but the yield of poly(L-diaminopropionic acid) was significantly lower compared with poly(ε-L-lysine). Thus, several low-cost carbon and nitrogen sources were screened as supplements. We tested glucose, wheat grain, rice bran, cane molasses, glycerol and corn flour as potential carbon sources (Figure 2(a)). The results showed that glucose was a suitable carbon source as observed in submerged fermentation, whereas industrial waste glycerol efficiently improved poly(ε-L-lysine) production with a yield comparable to glucose. In addition, the supplement of glycerol also increased the ratio PDAP/ε-PL, which resulted in a higher yield of poly(L-diaminopropionic acid), possibly caused by an influenced oxygen transfer and the biosynthesis of PDAP may prefer a lower oxygen level. Thus, industrial waste glycerol could be used as an optimal and economical supplement for high-yield production of both poly(L-diaminopropionic acid) and poly(ε-L-lysine) in solid-state fermentation. Although cane molasses are widely used as a raw material for the fermentation of microbial products and enzymes such as alcohol, succinic acid and proteases [34–36], this supplement was not optimal in the current study, possibly caused by an excess of metal ions.

Nitrogen sources also play a vital role in the biosynthesis of secondary metabolites. SO_4_^2-^ was indispensable for the production of poly(ε-L-lysine) in *Streptomyces*, where (NH_4_)_2_SO_4_ was used as a primary nitrogen source [28]. To screen a suitable organic nitrogen source, yeast extract, soybean meal, corn steep liquor and malt extract were used, with (NH_4_)_2_SO_4_ (3%, w/w) as positive control. Corn steep liquor produced comparable amounts of poly(L-diaminopropionic acid) and poly(ε-L-lysine) (Figure 2(b)), whereas when using also glycerol the yield of poly(L-diaminopropionic acid) remained high and the ratio PDAP/ɛ-PL reached 50%. This suggests consumption of methionine and cysteine in corn steep liquor since the thiol group is required for activation and polymerization of L-lysine or L-diaminopropionic acid. In contrast, using soybean meal and malt extract generated low levels of both products, possibly because of less available nutrition in the two components. Therefore, corn steep liquor and industrial waste glycerol were selected as optimal supplements for spent mushroom substrate in solid-state fermentation.

### 3.3 Optimization of solid-state fermentation conditions for the co-production of PDAP and ε-PL

#### 3.3.1 Optimization of initial moisture content

**Figure 3.**
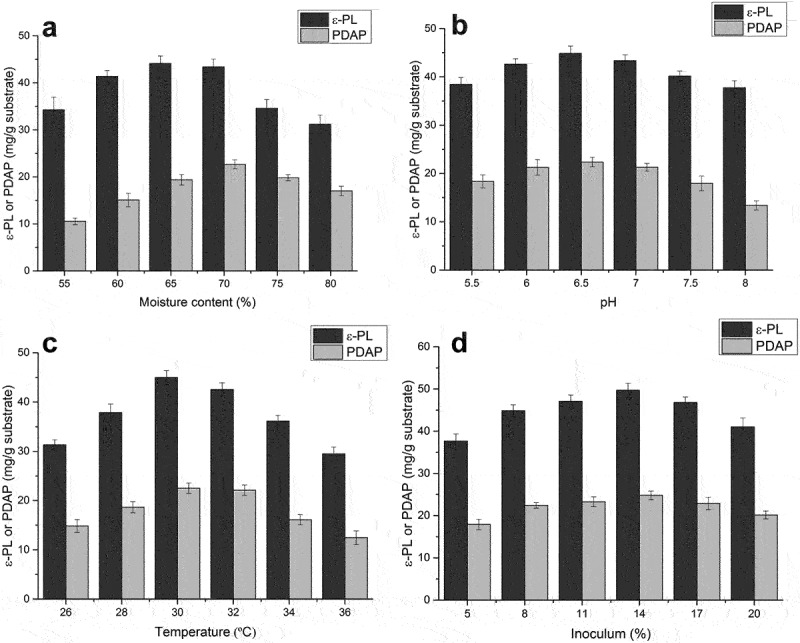
Optimization of fermentation conditions for co-production of poly(L-diaminopropionic acid) (PDAP) and poly(ε-L-lysine) (ε-PL). (a) Effect of initial moisture; (b) Effect of initial pH; (c) Effect of fermentation temperature; (d) Effect of inoculum level. Glycerol and corn steep liquor was used as carbon and nitrogen sources for optimization. Data points represent means (n = 3) ± standard deviation (SD).

In addition to nutritional factors, fermentation conditions also have a significant impact on the production of secondary metabolites, and therefore the effects of moisture content, initial pH, fermentation temperature and inoculum size were investigated. Moisture content of materials plays an important role in solid-state fermentation, with previous studies reporting that a high yield of the antibiotic meroparamycin in *Streptomyces* sp. was only achieved when the initial moisture was 60% [37] and maximal enzyme production was achieved at an initial moisture content of 71.43% [38]. The critical role of moisture content in solid-state fermentation may be related to the physical characteristics of the particles in the substrate; increasing moisture may reduce porousness, thereby limiting oxygen and mass transfer. Conversely, a lower moisture content may reduce the solubility of nutrients, resulting in less swelling and affecting the formation of secondary metabolites [39]. In the present study, we evaluated moisture contents ranges from 55% to 80% (Figure 3(a)). The highest production of poly(L-diaminopropionic acid) (22.7 mg/g substrate) and poly(ε-L-lysine) (44.1 mg/g substrate) was reached when moisture content was 70% and 65%, respectively. Considering that poly(ε-L-lysine) yield remained high, whereas the poly(L-diaminopropionic acid) production increased significantly, 70% was selected as the optimum moisture condition for co-production of these homopoly (amino acids). The initial moisture content had a significant effect on the ratio ε-PL/PDAP and more poly(L-diaminopropionic acid) was produced when moisture content was higher than 65%.

#### 3.3.2 Optimization of pH

Production of these homopoly (amino acids) was affected by pH. An acidic pH of 4.0 is required, possibly because of the regulation of enzyme catalysis in ε-PL or PDAP synthetase [40], but *S. albulus* grows optimally at pH 6.0. Thus, a two-stage pH control strategy seems preferable for fermentation. When the spent mushroom substrate was used for solid-state fermentation, the initial pH varied from 5.5 to 8.0 and the fermentation was not subject to pH control. Interestingly, the pH in the stationary phase was constant around 4.0, and the products were efficiently accumulated. Maximum yields of poly(ε-L-lysine) (44.8 mg/g substrate) and poly(L-diaminopropionic acid) (22.4 mg/g substrate) were achieved at pH 6.5 (Figure 3(b)). This was possibly caused by the application of corn steep liquor, which contributes to acidity, whereas glycerol maintains pH and productivity. Notably, an increase in pH above 7 led to a significant decrease in both PDAP production and PDAP/ε-PL ratio.

#### 3.3.3 Optimization of fermentation temperature

After optimizing the pH in the fermentation, the effect of temperature was investigated. Extremes of temperature adversely affect microbial metabolic activity [41]. We found that the optimum temperature was 30°C, whereas lower or higher temperatures result in lower yields (Figure 3(c)). The highest yield for poly(ε-L-lysine) and poly(L-diaminopropionic acid) was 45.0 mg/g substrate and 22.5 mg/g substrate, respectively. The ratio PDAP/ε-PL remained constant at lower temperatures, but poly(L-diaminopropionic acid) production was significantly affected at higher temperatures, especially above 32°C, suggesting that PDAP synthesis was more vulnerable to temperature.

#### 3.3.4 Optimization of inoculum level

The inoculum level was also important in fermentation, therefore various inoculum levels were analyzed to evaluate their effects. The highest yields of poly(ε-L-lysine) (49.7 mg/g substrate) and poly(L-diaminopropionic acid) (24.8 mg/g substrate) were obtained when the inoculum level was 14% (v/w) (Figure 3(d)). At lower or higher inoculum levels, production decreased. This can be explained by insufficient biomass when low levels of inoculum are used, which results in reduced product formation, whereas high levels of inoculum produce excessive biomass and depleted nutrients needed for product synthesis.

Overall, the optimal solid-state fermentation conditions for the co-production of poly(L-diaminopropionic acid) and poly(ε-L-lysine) using spent mushroom substrate were 70% initial moisture content, pH 6.5, 30°C and an initial inoculum size of 14%.

### 3.4 Fermentation amplification in 5 L flasks and repeated-batch fermentation

**Figure 4.**
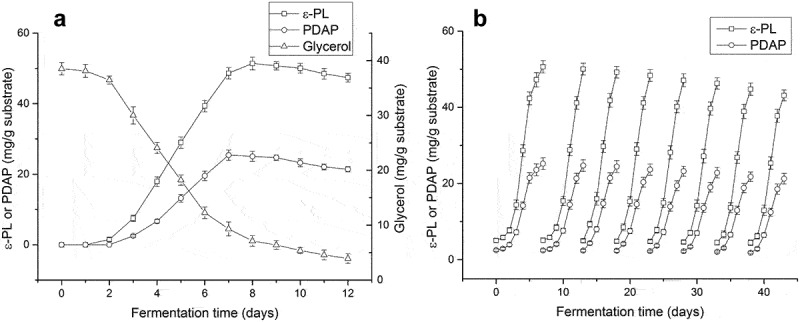
Time course of co-production of poly(L-diaminopropionic acid) (PDAP) and poly(ε-L-lysine) (ε-PL) under optimal fermentation conditions. (a) Amplified fermentation in 5 L flask; (b) Repeated-batch fermentation. Data points represent means (n = 3) ± standard deviation (SD).

After optimization of the fermentation conditions using spent mushroom substrate, amplified fermentation in 5 L flasks was investigated. The accumulation of poly(L-diaminopropionic acid) and poly(ε-L-lysine) was proportional to the consumption of glycerol (Figure 4(a)). The maximum yield of poly(ε-L-lysine) (51.4 mg/g substrate) was reached at day 8, whereas maximum production of poly(L-diaminopropionic acid) (25.4 mg/g substrate) was reached at day 7. After that, both products decreased gradually because of the depletion of glycerol and other nutrients. Since the accumulation of poly(L-diaminopropionic acid) decreased slightly at day 8, eight-day fermentation was selected to harvest these homopoly (amino acids).

Repeated-batch or continuous batch fermentation, is usually employed to reduce fermentation time in solid-state fermentation because it shortens the lag phase of microbial growth. For example, repeated-batch solid-state fermentation was used to produce natamycin continuously for five cycles [42], the L-tyrosine productivity and yield using *E. coli* were significantly increased by repeated batch fermentation compared with fed-batch fermentation [43]. In the present study, the lag phase of *S. albulus* growth took 2–3 days, and repeated-batch experiments were carried out to shorten the fermentation time and increase the specific time for biosynthesis of products. Poly(L-diaminopropionic acid) and poly(ε-L-lysine) were produced in eight fermentation cycles, and the fermentation time was reduced from 8 days to 6 days (Figure 4(b)). During continuous batch fermentation, the accumulation of these products was relatively stable. Compared with the 7 days yield in single batch solid-state fermentation, poly(ε-L-lysine) productivity was increased by 27.7% to a maximum of 8.2 mg/g substrate/day, and poly(L-diaminopropionic acid) productivity increased by 12.3%, to a maximum of 4.1 mg/g substrate/day. Therefore, the use of repeated-batch in solid fermentation of spent mushroom substrates is a feasible approach.

### 3.5 Application of PDAP and ε-PL to the biological preservation of food

**Figure 5.**
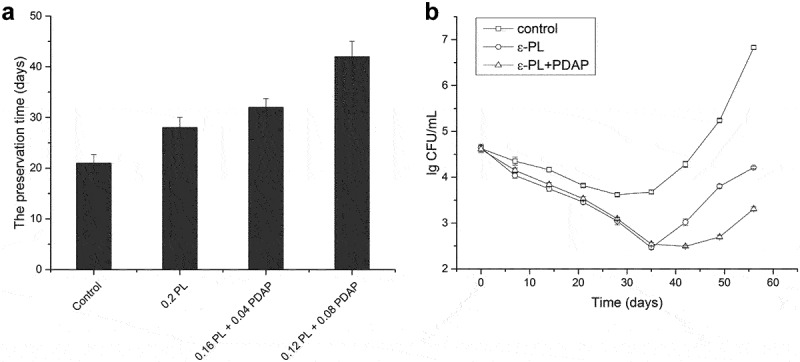
Application of poly(L-diaminopropionic acid) (PDAP) and poly(ε-L-lysine) (ε-PL) in the preservation of freshly pressed grape juice. (a) The preservation time of freshly pressed grape juice under different treatments. Control: Without treatment; 0.2 PL: treated with 0.2 g/kg ε-PL; 0.16 PL + 0.04 PDAP, treated with 0.16 g/kg ε-PL plus 0.04 g/kg PDAP; 0.12 PL + 0.08 PDAP, treated with 0.12 g/kg ε-PL plus 0.08 g/kg PDAP; (b) Microbial growth in freshly pressed grape juice during preservation under different treatments. Data points represent means (n = 3) ± standard deviation (SD).

The antimicrobial effects of the extracted poly(L-diaminopropionic acid) and poly(ε-L-lysine) were evaluated in food preservation experiments. Freshly pressed grape juice was used since its low pH and high sugar content favors the growth of yeast. In the control experiment, where no preservative was added, both flavor and color scores decreased dramatically after 3 weeks of storage. In contrast, grape juice treated with 0.12 g/kg ε-PL and 0.08 g/kg of PDAP increased its preservation time to 6 weeks and the addition of homopoly (amino acids) at this amount had no obvious effect on the taste, whereas treatment with ε-PL only extended the shelf life one week more than the control (Figure 5(a)). Poly(ε-L-lysine) and poly(L-diaminopropionic acid) are natural antimicrobial peptides, their antimicrobial activity depends on the unique cationic structures. The mechanisms involved in destabilizing membranes by interacting with negatively charged phospholipid head groups, accumulation of reactive oxygen species and DNA fragmentation in microorganisms [44–46]. However, it has been reported that the antibacterial activity of poly(ε-L-lysine) could be limited to the food matrix because of their charged amino groups form complexes with food polyanions [47]. In the present study, poly(ε-L-lysine) exhibited strong antibacterial activity in grape juice in the first 5 weeks indicating that the antibacterial activity of poly(ε-L-lysine) may not be limited in this food matrix, but after that yeasts started to grow rapidly. In contrast, the poly(ε-L-lysine) plus poly(L-diaminopropionic acid)-treated sample was better preserved (Figure 5(b)). Previous studies reported that the combination of poly(ε-L-lysine) and nisin showed synergistic antimicrobial activity, which revealed a promoted uptake of poly(ε-L-lysine) into cells [48]. Thus, the antimicrobial activity observed in the current study could be contributed by the stronger inhibitory activity against yeast of poly(L-diaminopropionic acid) and the synergistic antimicrobial activity of the two antimicrobial peptides.

## 4. Conclusion

In this study, the co-production of poly(L-diaminopropionic acid) and poly(ε-L-lysine) was achieved using spent mushroom substrate in solid-state fermentation. Through supplementation of glycerol and corn steep liquor and optimization of fermentation parameters, the yield of poly(ε-L-lysine) and poly(L-diaminopropionic acid) reached 51.4 mg/g substrate and 25.4 mg/g substrate, respectively. Repeated-batches of solid-state fermentation indicate that these products could be constantly co-produced with high efficiency. In addition, the combined application of poly(L-diaminopropionic acid) and poly(ε-L-lysine) exhibited a good potential as biological preservative. Thus, this fermentation strategy not only improves production but also enables the recycling of waste biomass and product diversification.

## Supplementary Material

Supplemental Material
