# Supplementary material for: Poly(ε-L-lysine) and poly(L-diaminopropionic acid) co-produced from spent mushroom substrate fermentation: potential use as food preservatives
Source: Bioengineered. 2022 Feb 21;13(3):5892–902. doi: 10.1080/21655979.2022.2040876 (PMC8973980; doi:10.1080/21655979.2022.2040876)
Supplement: Supplemental Material [file KBIE_A_2040876_SM4871.zip › supplementary/Supplementary material Table S1.docx]

Table S1 Mineral elements content in the spent mushroom substrate (mg/ 100g dry weight)

| K | P | Ca | Mg | Na | Fe | Zn | Mn |
| --- | --- | --- | --- | --- | --- | --- | --- |
| 1250±92 | 360±17 | 1680±102 | 340±35 | 120±16 | 75±7.6 | 12.7±1.0 | 19.5±2.8 |
