# Supplementary figures and images for: Poly(ε-L-lysine) and poly(L-diaminopropionic acid) co-produced from spent mushroom substrate fermentation: potential use as food preservatives
Source: Bioengineered. 2022 Feb 21;13(3):5892–902. doi: 10.1080/21655979.2022.2040876 (PMC8973980; doi:10.1080/21655979.2022.2040876)

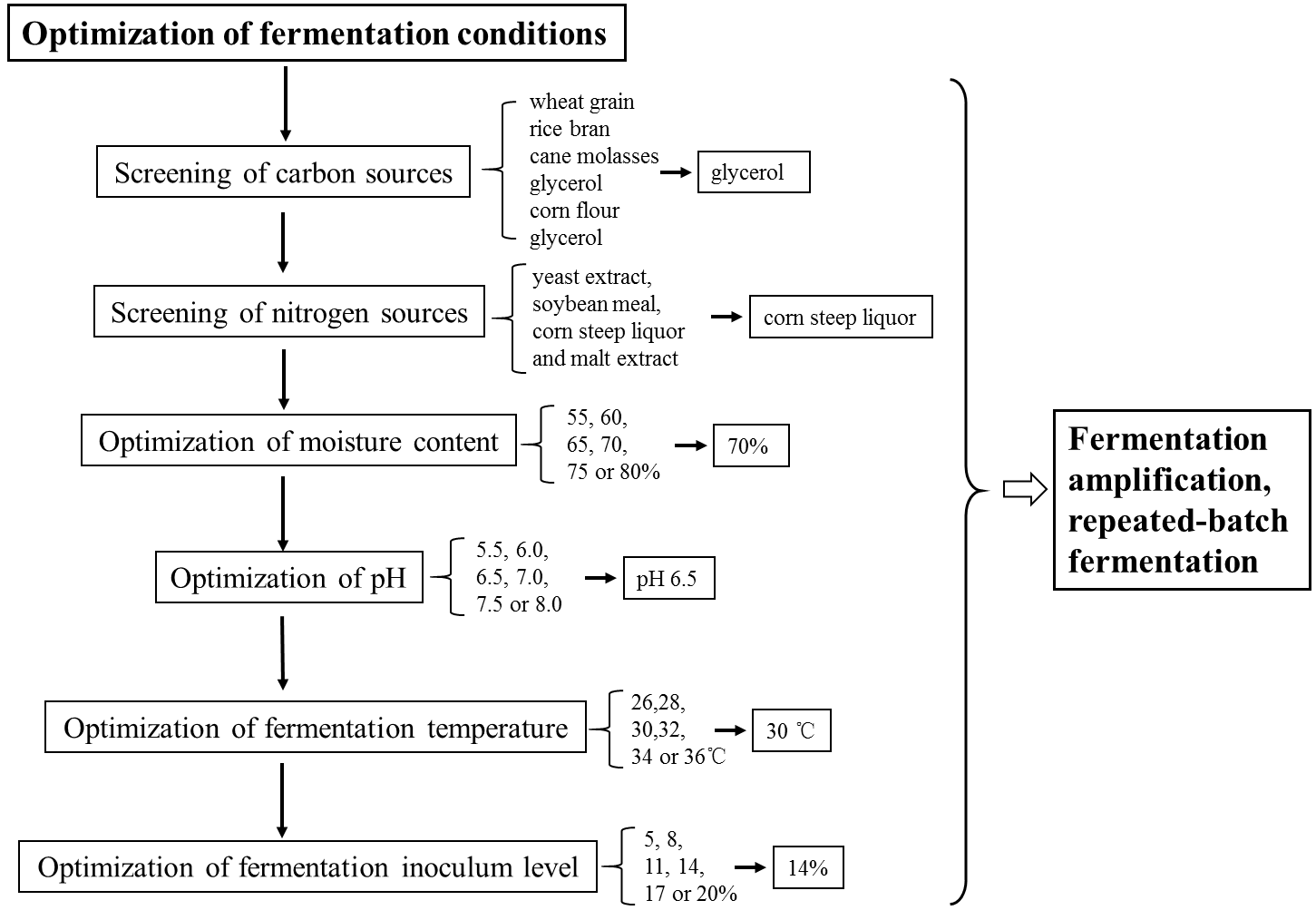


Fig. S1. Illustration of the experiment design for optimization of fermentation conditions.

Supplement: Supplemental Material [file KBIE_A_2040876_SM4871.zip › supplementary/Supplementary material Figure S1.docx]
